# Solid state NMR - An indispensable tool in organic-inorganic biocomposite characterization; refining the structure of octacalcium phosphate composites with the linear metabolic di-acids succinate and adipate

**DOI:** 10.1016/j.ssnmr.2018.08.004

**Published:** 2018-11

**Authors:** Yang Li, David G. Reid, Melinda J. Duer, Jerry C.C. Chan

**Affiliations:** a Department of Chemistry, University of Cambridge, Lensfield Road, Cambridge, Cambs, CB2 1EW, United Kingdom; b Department of Chemistry, National Taiwan University, College of Science, No. 1, Section 4, Roosevelt Road, Taipei, 10617, Taiwan

**Keywords:** Adipate, Biocomposite, Biomineralization, Hydroxyapatite precursor, OCP, Octacalcium phosphate, PDSD, REDOR, Succinate

## Abstract

Octacalcium phosphate (OCP; Ca_8_(HPO_4_)_2_(PO_4_)_4_. 5H_2_O) is a plausible precursor phase of biological hydroxyapatite, which composites with a number of biologically relevant organic metabolites. Widely used material science physicochemical structure determination techniques successfully characterize the mineral component of these composites but leave details of the structure, and interactions with mineral, of the organic component almost completely obscure. The metabolic linear di-acids succinate (SUC) and adipate (ADI) differentially expand the hydrated (100) layer of OCP. ^13^C—^13^C correlation (proton driven spin diffusion, PDSD) experiments on OCP composited with (U-^13^C_4_)-SUC, and (U—^13^C_6_)-ADI, show that the two di-acids per unit cell adopt non-centrosymmetric but mutually identical structures. ^13^C{^31^P}, rotational echo double resonance (REDOR) shows that one end of each linear di-acid is displaced further from the surface of the apatitic OCP layer relative to the other end. Overall the results indicate two di-acids per unit cell disposed perpendicularly across the OCP hydrated layer with one carboxylate of each di-acid substituting a hydrated surface OCP phosphate group. This study re-affirms the unique advantages of ssNMR in elucidating structural details of organic-inorganic biocomposites, and thereby mechanisms underlying the roles of small metabolites in influencing biomineralization mechanisms and outcomes.

## Highlights

• OCP-succinate and OCP-adipate, are well ordered and amenable to NMR structure refinement.
• DSD of ^13^C enriched di-acids reveal non-centrosymmetric conformers.
• ^13^C{^31^P} REDOR shows asymmetric placement with respect to OCP hydrated surface, yielding valuable structural constraints.

## Graphical abstract

**Figure:**
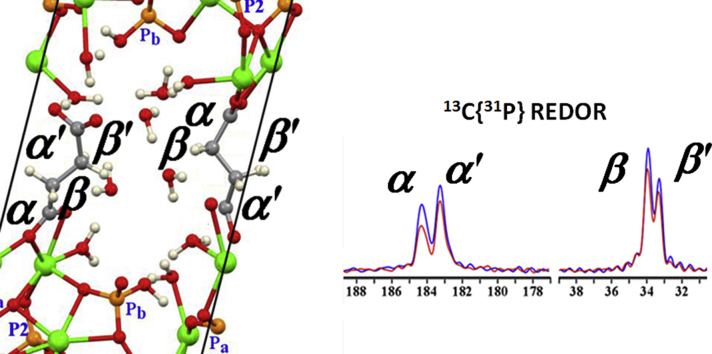

## 1 Introduction

Bone is a complex hydrated organic-inorganic composite material. The networks of organic matrix, mineral, and hydrating water, are impossible to isolate without disruption of their respective structures. Deproteination and microscopic fixation can destroy the delicate bio-apatite mineral platelet structure by dehydration, with collapse into more aggregated forms [1]. Moreover, bone micro- and nano-structure varies considerably due to the individual organism, age, species, anatomical site, and pathological and metabolic status. It is well established that citrate is frequently a significant component of mammalian bone mineral [[2], [3], [4]], and other small metabolites such as lactate can also be, and frequently are, incorporated [5]. It is conceivable that different bone samples may have different metabolite incorporations in the mineral, which could contribute to variations in material and chemical properties such as hardness, solubility, and re-absorption. Therefore, well chosen model calcium phosphate – small molecule composites, which can be synthesized with well defined homogeneous compositions in sufficient quantities for detailed structural analysis, can help elucidate how the incorporation of organic metabolites may affect biomineral structure and physical properties.

Octacalcium phosphate (OCP; Ca_8_H_2_(PO_4_)_6_·5H_2_O) is frequently invoked to model the precursor structures of mature hydroxyapatitic biomineral, possessing as it does apatite-like layers “sandwiching” layers of hydrated calcium phosphate/hydrogen phosphate [6,7], and the propensity to convert readily to the more thermodynamically stable hydroxyapatite [8]. OCP-succinate (OCP-SUC) [9] has attracted interest, partly because its facile preparation and highly crystalline nature make structural characterization, at least of the mineral component, (e.g. by powder XRD and FTIR [[10], [11], [12], [13]]) feasible, thereby shedding light on fundamental molecular mechanisms and recognition processes underlying the participation of small metabolites in biomineralization. Many different metabolites can coordinate to calcium, and it is possible others besides those already mentioned are incorporated within native biomineral structures [2,3,[14], [15], [16]]. Due to differences in size, stereochemistry, calcium affinity, and acidity, incorporation of different metabolites will yield minerals with different physical and chemical properties. Besides succinate, itself a citric acid cycle intermediate, OCP composites incorporating numerous other organic acids, many of them important metabolites often present in significant concentrations in mineralizing tissue, have been synthesized and characterized by XRD, IR and Raman spectroscopy [[17], [18], [19]]. Though these techniques quantify the organic component and changes in mineral crystal structure, they provide little information on the geometric relationship of the incorporated organic acids to the host mineral matrix beyond changes in unit cell dimensions, or of the conformational and structural properties of the organic component itself [18]. Tsai et al. [10] have shown by solid-state NMR (ssNMR) that succinate anions in OCP-SUC substitute certain of the phosphate sites of OCP [20,21], although a later study [22] used ssNMR and density functional theory (DFT) calculations to revise some of the original OCP ^31^P NMR assignments. OCP-citrate (OCP-CIT) has been proposed as a model for the hydrated layers between bone mineral platelets in which citrate anions coordinate calcium ions and bridge the hydrated layer between apatitic sheets [23].

Towards understanding the participation of other metabolic acids in native biomineralization we have synthesized and characterized OCP-SUC, and its six-carbon dicarboxylic acid homologue OCP-adipate (OCP-ADI), as model compounds and used ^31^P and ^13^C ssNMR to refine our understanding of their structures.

## 2 Materials and methods

### 2.1 Synthesis of OCP, OCP-SUC, and OCP-ADI

Most reagents were purchased from Sigma-Aldrich and used as received. (U-^13^C_4_)-SUC and (U—^13^C_6_)-ADI were purchased from Cambridge Isotope Laboratories (Andover, MS, USA).

The relevant organic acid was dissolved in water (200 ml) and the pH was adjusted to 5.5 by dropwise addition of NaOH. The solution was heated to 60 °C with stirring, and calcium carbonate (1.604 g, 16 mmol) and orthophosphoric acid (0.6 ml, 10 mmol) were added. The suspension was left to stir for 5 h, and the solid was collected by gravity filtration and dried in air to a white powder. Reagent mass and volumes were reduced, as below, for the synthesis of the isotope enriched materials. Although the proportions of the reactants may have differed from those of each functional group in our products, the thermodynamic drive to produce OCP itself, and OCP-SUC and OCP-ADI composites of reproducible stoichiometry must be considerable, with excess ions remaining in solution.

Reagent and solvent concentrations, and mean product yields, were: Pure OCP (no organic acid added, and no pH adjustment required) collected as white powder (1.14 g, 0.58 mmol). OCP-SUC (2.363 g, 20 mmol) collected as white powder (1.25 g). OCP-ADI (2.92 g, 20 mmol) collected as white powder (1.29 g). OCP-(U-^13^C_4_)-SUC (^13^C labelled succinic acid 60 mg, 0.492 mmol) collected as white powder (29.4 mg). OCP-(U—^13^C_6_)-ADI (^13^C labelled adipic acid 73 mg, 0.492 mmol) yield 33 mg. Molar yields were calculated from microanalysis data (see below) by assuming 16 calcium and eight (PO_4_)^3−^ions per unit cell, that all carbon originated from the organic acids, and that hydrogen in excess of that appropriate to the organic acid and (HPO_4_)^2-^ content corresponded to water.

Elemental analysis was performed by the Microanalysis service, Department of Chemistry, University of Cambridge. The CHN elements were measured with an Exeter Analytical CE440 elemental analyser with samples combusted at 975 °C in oxygen. Calcium and phosphorus were measured with a Thermo Scientific 7400 ICP-OES, at 396.84 nm and 178.28 nm respectively. Samples were characterized by powder X-ray diffraction (XRD) at room temperature on a PANalytical Empyrean diffractometer in Bragg−Brentano geometry using Cu Kα radiation.

### 2.2 NMR spectroscopy

A Bruker 400 MHz Avance spectroscopy II spectrometer was used for solid-state ^1^H, ^13^C and ^31^P NMR measurements, at frequencies of 400.42 MHz, 100.6 MHz and 162.1 MHz respectively, with standard Bruker double and triple resonance MAS probes. Samples were packed into disposable HR-MAS inserts where necessary, and 4 mm zirconia rotors for magic angle spinning (MAS) at 10 kHz.

Samples were characterized initially using ^31^P direct-polarisation (DP), and ^13^C and ^31^P cross-polarisation (CP) (^1^H 90° pulse length 2.5 μs, ^31^P 90° pulse length 2.6 μs, ^1^H—^13^C CP contact time 2500 μs, ^1^H—^31^P CP contact time 10 ms, recycle times 600 s for ^31^P DP experiments, and 2 s for CP experiments and all experiments described subsequently which were initiated using CP. The ^1^H—^31^P heteronuclear correlation (HETCOR) experiments were performed with frequency-switched Lee-Goldburg (FSLG) decoupling during t_1_ (^1^H field strength 100 kHz, 2 ms contact time). The POST-C7 [24] pulse sequence was used for the 2D single quantum-double quantum (SQ-DQ) ^31^P correlation experiments (^31^P field strength 61 kHz, 28 composite C7 cycles for DQ excitation and reconversion). For the PDSD experiments a 3.6 μs ^13^C 90° pulse was used to return magnetization to the z-axis of the rotating frame after initial cross polarisation, for a period during which ^1^H — ^1^H spin diffusion in the absence of broadband decoupling transferred magnetization between neighbouring ^13^C's, before ^13^C magnetization was returned to the x,y-plane with a second 90° pulse for detection. REDOR experiments were carried out using a train of rotor-synchronized ^31^P 5.2 μs 180° pulses to recouple ^31^P — ^13^C dipolar interactions with a 7.2 μs ^13^C refocussing pulse, and REDOR dephasing times of 2–10 ms. Broadband SPINAL 64 ^1^H decoupling at a field strength of 70 kHz was applied during signal acquisition for all the above experiments. ^13^C spectra were referenced to the glycine Cα signal at 43.1 ppm relative to TMS at 0 ppm. ^31^P spectra were referenced to the hydroxyapatite signal at 2.6 ppm relative to 85 wt% H_3_PO_4_ at 0 ppm.

## 3 Results and discussion

### 3.1 Synthesis and initial characterization

**Fig. 1:**
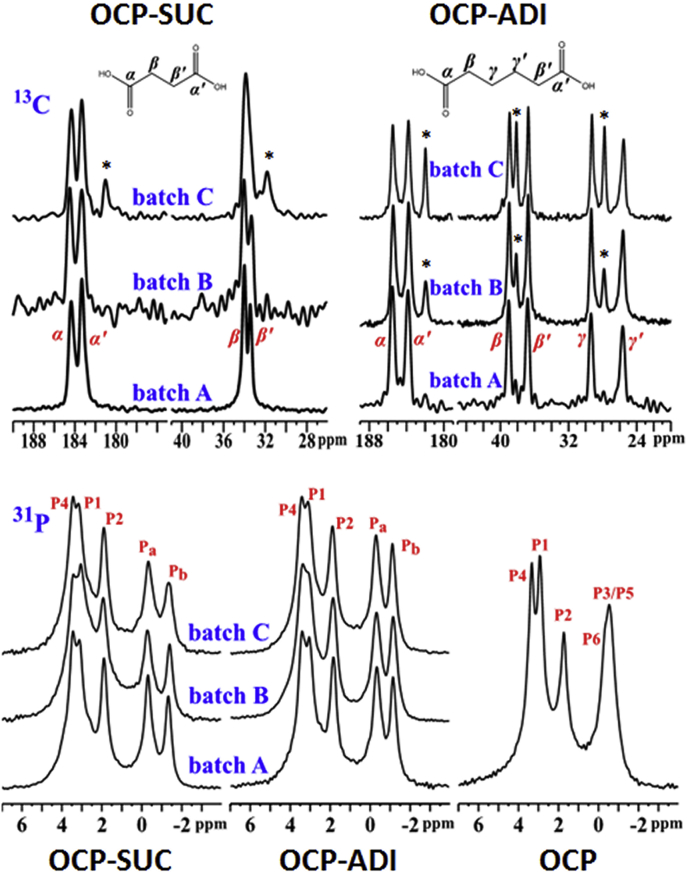
Comparison between ^13^C, and ^31^P, NMR spectra of different batches of OCP-SUC and OCP-ADI, and pure OCP. Minor ^13^C signals in some batches, of variable occurrence, are marked by asterisks.

Synthesis and initial characterizations of OCP, OCP-SUC, and OCP-ADI, including elemental analysis (Electronic Supplementary Information, ESI, Table. S1), powder XRD (Fig. S1), and ^31^P NMR, essentially replicated literature procedures and results, and are summarized and discussed in the ESI. Empirical formulae were calculated as described in Materials and Methods and were in excellent agreement with the data of Markovic et al. [18] (OCP-SUC Ca_8_(PO_4_)_4_(HPO_4_)_1.08_ (succinate)_0.91_ · 5–6H_2_O; OCP-ADI Ca_8_(PO_4_)_4_(HPO_4_)_1.27_ (adipate)_1.1_ · 5–6H_2_O Elemental compositions confirm that OCP-SUC and OCP-ADI both have two acid molecules per unit cell i.e. per sixteen calcium atoms. Carbon contents slightly above (ca. 10%) theoretical can be accounted for by carbonate incorporation (not quantified) and some metabolite attachment to crystal surfaces. OCP-SUC and OCP-ADI should have very similar structures, with adipates substituting the P5 phosphate group [7] and oriented analogously to the succinates in OCP-SUC. ^31^P spectra of different batches of both composites are very similar to each other, and to ^31^P data already published for OCP [20,22] and OCP-SUC [10] (Fig. 1) including 2D SQ-DQ ^31^P — ^31^P (Fig. S2) and ^1^H–^31^P HETCOR (Fig. S3) correlation experiments. There is some interbatch variability in the ^13^C spectra, which are shown in Fig. 1 along with the corresponding ^31^P spectra, suggesting that a minor proportion of the organic acids can occupy alternative environments in the hydrated layer, likely due to interactions with variable water molecule numbers and/or locations, and acid orientations, but whatever these are it is clear they leave the phosphorus environments practically unaffected. Indeed calculations from XRD of the mean d_100_ interlayer spacing in five batches of OCP-SUC showed very little interbatch variation in this parameter (mean 2.136 nm, standard deviation 0.024 nm equivalent to only 1.12% of the mean). The sharpness of the ^31^P signals, and the similarity of the OCP and composite spectra, indicate similar symmetry within the crystallographic unit cell, which is P1 in the case of OCP [7]. This symmetry can be retained when either of the symmetric di-acids is substituted into the hydration layer. The (generally) sharp ^13^C peaks also support the idea that the acids are ordered within the composite structure; that only two major signals for each of the four OCP-SUC, and four for each of the eight OCP-ADI, methylene carbons, and two signals for each of the four carboxylate, environments possible per unit cell implies unit cell symmetry. The sharpness and the chemical shifts of Pa and Pb ^31^P NMR signals suggests little proton exchange via OCP hydrogen bond breaking and re-forming, and that the water environments are different for these two phosphates. On the basis of their chemical shifts Pa signals are from hydrated orthophosphates, and Pb from hydrogen phosphates also hydrogen bonded to water molecules. This inference is in agreement with more detailed arguments [10] which are based on the much larger NMR chemical shift anisotropy of HPO_4_^2−^ (Pb) relative to that of the more symmetrical PO_4_^3−^ ion.

### 3.2 Structural properties of the organic di-acids: ^13^C—^13^C PDSD correlation

**Fig. 2:**
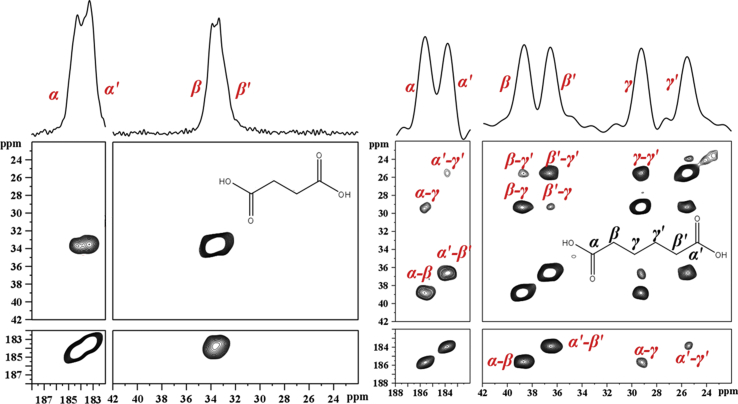
^13^C—^13^C proton-driven spin diffusion correlation (PDSD, 5 ms mixing time) experiments on uniformly ^13^C labelled OCP-SUC (left) and OCP-ADI (right). Correlations proving a single non-centrosymmetric ADI within the OCP-ADI structure are highlighted in red. Corresponding 1D ^13^C spectra are superimposed. Signal broadening relative to unlabelled composites is ascribed to residual ^13^C—^13^C dipolar interactions, and unresolved ^13^C—^13^C scalar coupling. (For interpretation of the references to colour in this figure legend, the reader is referred to the Web version of this article.)

^13^C NMR spectra of OCP-SUC and OCP-ADI indicate there are two predominant environments for the carboxylate, and for the methylene, carbons. To establish whether these peaks are due to a single non-centrosymmetric, or two inequivalent but centrosymmetric organic acid structures, OCP-(U-^13^C_4_)-SUC and OCP-(U—^13^C_6_)-ADI were synthesized, and 2D^13^C—^13^C homonuclear PDSD correlation experiments were performed, shown in Fig. 2. In the ^13^C-labelled OCP-SUC spectral resolution is insufficient to extract information about the connectivity (or otherwise) between the methylene carbons, so whether the signals are from one SUC molecule or two cannot be determined by this experiment.

On the other hand the ^13^C spectrum of OCP-ADI is disperse enough that clear PDSD correlations are evident at short mixing times between the two low frequency signals corresponding to the two central methylene carbons (Cγ 29.4 ppm and Cγ′ 25.7 ppm), which are therefore most likely bonded to each other as well as structurally inequivalent. All the other correlations in Fig. 2 (e.g. between Cβ 38.7 ppm and Cγ′, and Cβ′ 36.6 ppm and Cγ) are also consistent with two identical but non-centrosymmetric ADI structures per unit cell. We also considered two other possibilities: firstly that the PDSD effects might be intermolecular, i.e. between intercalated non-equivalent but centrosymmetric di-acids, or secondly that the PDSD effects may be mediated by interconversion between two conformers, again non-equivalent but centrosymmetric. We discount the first possibility as unlikely because no magnetization transfer is observed between Cα and Cγ′, or between Cα′ and Cγ, although it is observed between Cβ and Cβ'. If the latter is intermolecular it is highly likely that the former would be observed as well, but they are not. We discount the second possibility i.e. that the α site is undergoing conformational exchange with the α′ site, and so on, because the exchange if it takes place must be slow on the ^13^C chemical shift time scale for distinct, and not single time-averaged, signals to be seen. In the OCP-SUC the smallest frequency separation is between β and β′ carbon signals and is ca. 0.5 ppm i.e. 50 Hz. This implies that if β and β′ are exchanging with each other the lifetime of each state is much greater than 20 ms. The corresponding smallest separation (α and α′) in OCP-ADI is 175 Hz implying a residence time in each state of well over 6 ms. The PDSD mixing time is only 5 ms i.e. much shorter than the mean residence time, so cross peaks Cβ – Cβ′, and Cγ – Cγ′, are unlikely to be exchange mediated, nor do we see any Cα – Cα’ exchange.

### 3.3 Mineral-organic phase interactions: ^13^C{^31^P} REDOR

**Fig. 3:**
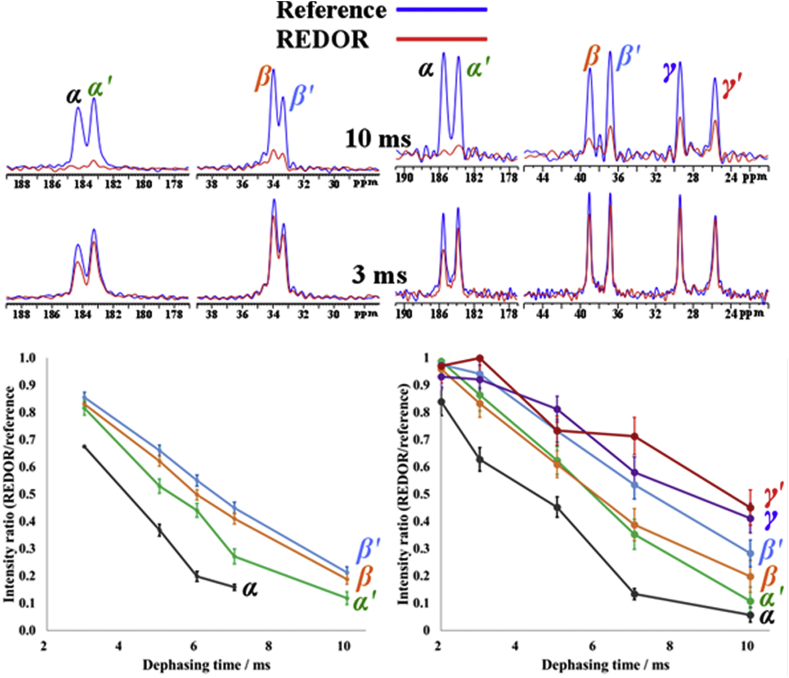
Typical ^13^C{^31^P} REDOR data (top) for OCP-SUC (left) and OCP-ADI (right), and resultant dephasing curves (bottom), showing the non-equivalence of the carboxylate carbons, and of the methylene carbons, with respect to neighbourhood phosphorus atoms Error bars in the dephasing curves represent spectral signal-to-noise measurements at each respective dephasing time.

The ^13^C{^31^P} heteronuclear dipole-dipole recoupling REDOR [24] experiment is a particularly powerful technique in structure characterization of phosphatic biomineral as it reports the spatial relationships and proximities between carbon (i.e. the organic acids in this case) and phosphorus (i.e. the mineral phase) atoms within the organic-inorganic composites [4,25]. In the ^13^C{^31^P} REDOR spectra for OCP-SUC and OCP-ADI (Fig. 2) all the di-acid carbon signals diphase when the ^31^P—^13^C dipole-dipole interaction is re-introduced, but to different extents. This dephasing signal loss is greater the closer a carbon atom is to its phosphorus neighbours. This is direct confirmation that both acids are incorporated inside the OCP structure, and suggests that the two carboxylate ^13^C environments also have different effective distances to neighbouring phosphorus atoms. Plotting signal intensity change relative to reference spectra against REDOR dephasing time produces dephasing curves (Fig. 3) which confirm a range of carbon dephasing rates in both composites, with carboxylate signals dephasing faster than those of the methylene carbons. This is consistent with chelation of acid carboxylates to calcium ions in the OCP apatitic layer which shows they are closer to phosphorus atoms than the methylene carbons are. The difference in dephasing rates in both OCP-SUC and OCP-ADI suggest one carboxylate is closer to mineral than the other. Considering all the information, the OCP-SUC and OCP-ADI structures should have the following features: Similar phosphorus sites to OCP; P5 phosphate groups substituted by organic acid carboxylates; a unit cell with a centre, or approximate centre, of symmetry; two structurally near-identical non-centrosymmetric di-acids per unit cell; and carboxylate-phosphorus distances different for the different carboxylate environments corresponding to the two 13C chemical shifts.

### 3.4 Refining models of OCP-SUC and OCP-ADI structures

**Fig. 4:**
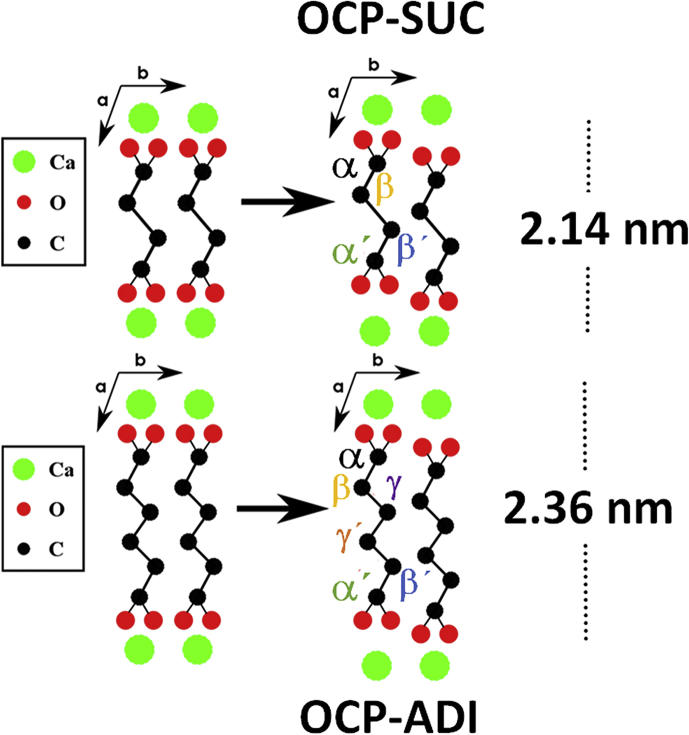
Diagrams on the right depict structures of OCP-SUC (top) and OCP-ADI (bottom) consistent with two identical non-centrosymmetric di-acids per unit cell, modified from those implied by Model I of Markovic et al. [18], (left) which implies the existence of two identical and centrosymmetric di-acids.

Considering all the data, and our structural models of OCP-citrate [23], we propose very similar models of OCP-SUC and OCP-ADI (Fig. 4), with two di-acid molecules per unit cell substituting two HPO_4_^2−^ sites (thus retaining charge balance), and oriented vertically across the hydrated layer. The two carboxylate groups on each di-acid are at different distances from the apatitic layer, consistent with the REDOR dephasing. It is probable that the more rapidly dephasing carboxylate (α in our nomenclature) is the one substituting the hydrogen phosphate ion and thus effectively buried in the surface of this layer. As well as their possible relevance to native biomineralization processes the high crystallinity of the OCP-SUC and OCP-ADI composites makes them particularly favourable systems for the study of more fundamental principles governing the role of small organic metabolites in healthy and pathological calcification.

## 4 Conclusion

Standard physicochemical tools for characterizing the structures of organic-inorganic biomimetic composites (elemental analysis, diffraction, vibrational spectroscopy, thermal methods, microscopy) provide an almost complete picture of the inorganic phases of OCP-SUC and OCP-ADI, revealing the expansion of the hydrated layer in proportion to the length of the included di-acid, and predicting the substitution of an OCP phosphate (although not which one) by an acid carboxylate. This expansion formed the basis of one of the models of OCP-SUC proposed by Markovic et al. [18]. Our ^13^C data clearly rule out this paper's Model II which invokes two non-equivalent di-acids per unit cell, while our ^13^C{^31^P} REDOR experiments show that the Model I is an oversimplification which implies equivalent interactions with the apatitic phase surface on the part of both carboxylates of each di-acid. Being able to establish these constraints on the organic components and their interface with the mineral makes ssNMR a uniquely versatile tool in the characterization of biomineral structure.

## Conflicts of interest

There are no conflicts to declare.

## Appendix A Supplementary data

OCP-SUC ADI Supplementary OCP-SUC ADI Supplementary
