# Supplementary material for: Solid state NMR - An indispensable tool in organic-inorganic biocomposite characterization; refining the structure of octacalcium phosphate composites with the linear metabolic di-acids succinate and adipate
Source: Solid State Nucl Magn Reson. 2018 Nov;95:1–5. doi: 10.1016/j.ssnmr.2018.08.004 (PMC6181798; doi:10.1016/j.ssnmr.2018.08.004)
Supplement: OCP-SUC ADI Supplementary [file mmc1.docx]

**Online Supplementary Information**

**Solid state NMR - an indispensable tool in organic-inorganic biocomposite characterization; refining the structure of octacalcium phosphate composites with the linear metabolic di-acids succinate and adipate.**

Yang Li,^a^ David G. Reid,^a^ Melinda, J. Duer,*^a^ and Jerry C. C. Chan^b^

a - Department of Chemistry, University of Cambridge, Lensfield Road, Cambridge, Cambs. CB2 1EW, United Kingdom.

b - Department of Chemistry, National Taiwan University, College of Science, No. 1, Section 4, Roosevelt Road, Taipei 10617, Taiwan.

* - Author for correspondence. E-mail [mjd13@cam.ac.uk](mailto:mjd13@cam.ac.uk) Telephone +44-1223-763

**OCP-SUC and OCP-ADI supplementary: Contents**

**Table S1**: Elemental and unit cell compositions and d_100_ spacings of OCP-SUC and OCP-ADI **P. 2**

**Fig. S1**. PXRD of OCP, OCP-SUC and OCP-ADI, and discussion of published models. **P. 2**

**Fig. S2**. Comparison of 2D ^31^P-^31^P SQ-DQ correlation spectra of OCP-SUC, OCP-ADI, and pure OCP. **P. 3**

**Fig. S3**. Comparison of ^1^H-^31^P HETCORs of OCP-SUC, OCP-ADI, and pure OCP, and discussion. **P. 4**

**References**  **P. 4**

**Table S1** Mean elemental composition, corresponding numbers of carbon and phosphorus atoms per unit cell (based on 16 calcium ions), and d_100_ spacing (c.f. pure OCP d_100_ = 18.76 Å)

| Compound | Elemental content (Wt %) | | | | C/Unit cell | P/Unit cell | d_100_/Å |
| --- | --- | --- | --- | --- | --- | --- | --- |
|  | C | H | Ca | P |  |  |  |
| OCP-SUC | 4.26 | 1.48 | 31.60 | 15.13 | 8.7 | 9.9 | 21.36 |
| OCP-ADI | 6.11 | 1.77 | 30.09 | 14.45 | 13.1 | 9.9 | 23.59 |


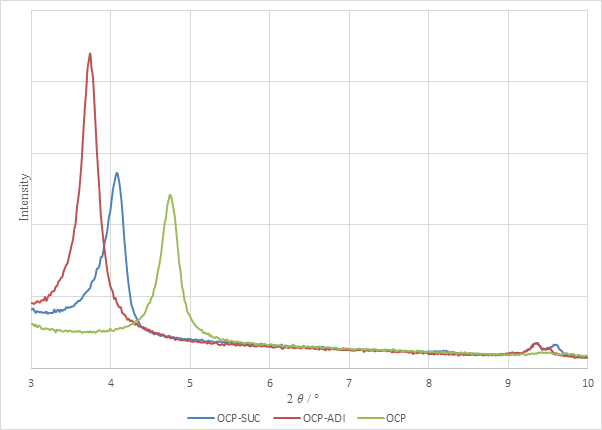


**Figure S1** Detail from the powder X-ray diffractograms of pure OCP, and OCP-SUC and OCP-ADI.

The shift in the powder XRD (100) reflection of OCP-SUC and OCP-ADI (Fig. S1) is direct evidence of the expansion of the OCP unit cell along the a-axis to accommodate the incorporation of the organic acids. The measured (100) reflection angles correspond to d­_100_ spacings of 1.876 nm for OCP, 2.136 nm for OCP-SUC, and 2.359 nm for OCP-ADI, also shown in Table S1, consistent with those reported by Markovic et al. [1]. The number of phosphorus atoms per unit cell shows that OCP-SUC and OCP-ADI have two phosphate groups replaced by carboxylate groups. The difference in the d_100_ spacing between OCP-SUC and OCP-ADI is 2.24 Å, comparable to the length of two methylene carbon units (2.5 Å), suggesting that the succinates and adipates have very similar orientations within the structure.

These formulae predict two (succinate)^2-^, and two (adipate)^2-^, species within the respective unit cells of each composite, based on which two models of OCP-SUC have been proposed [1]; *Model I* places both succinates vertically across the hydration layer of OCP, while *Model II* proposes one succinate vertically across the hydration layer with the other diagonal. The diagonal distance of the hydration layer in the OCP unit cell is around 0.98 nm, and the length of succinate is around 0.526 nm, while the average Ca-O bond distance is 0.25 nm between succinates and calcium ion. The substitution in *Model II* [1] will not yield the sizeable expansion (predicted 0.26 nm increase) in the a-axis detected experimentally. Therefore, *Model I* is used as a starting point to refine the structure of OCP-SUC.


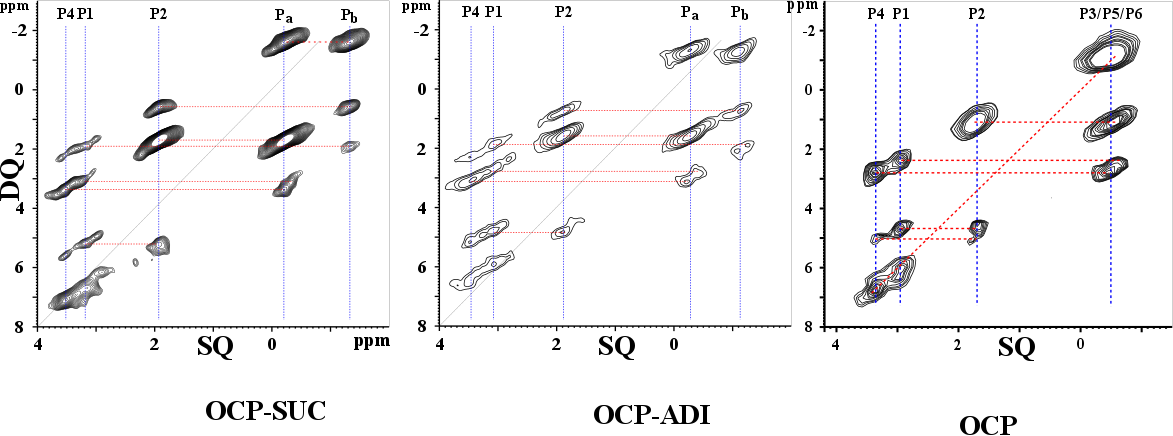


**Figure S2** A comparison of 2D SQ-DQ 31P – 31P correlation spectra for OCP-SUC, OCP-ADI, and pure OCP.

2D ^31^P single quantum – double quantum (SQ-DQ) correlation NMR data show the spatial proximities of pairs of ^31^P nuclei; again very similar to published spectra, and indeed to each other showing the same pattern of correlations in the orthophosphate region (P4/P1/P2 using the crystallographic phosphorus atom naming system of Mathew et al. [5]). However, the P1 orthophosphate self-correlation signal, resulting from spatial proximity of P1 in neighbouring unit cell is slightly different for OCP, and the composites. For OCP, the correlation falls as expected on the spectrum diagonal (y = 2x); for the composites, the corresponding correlation is slightly off-diagonal, suggesting slightly different P1 phosphorus environments between adjacent unit cells. This may be due to different acid orientations in different unit cells, or the acid orientation in each unit cell is not perfectly symmetric w.r.t the unit cell centre of symmetry, resulting in differences between the top and bottom apatitic layers within a unit cell, and hence two slightly different P1 phosphorus atoms in each unit cell. The major difference between the spectra for OCP and the composites is the separation of the single P3/P5/P6 correlation into two distinct Pa (P3) and Pb (P6) correlations. Despite this separation, all the correlations between P4/P1/P2 and P3/P5/P6 in OCP remained as correlations between P4/P1/P2 and Pa/Pb in the composites. The same correlation pattern between the orthophosphates and hydrogen phosphates for OCP and OCP-SUC confirms the idea that the orthophosphates in the apatitic region are not much affected by succinate incorporation and succinate substitutes hydrogen phosphate in the hydration layer, which very likely to be the OCP P5 phosphate due to the disappearance of this signal.


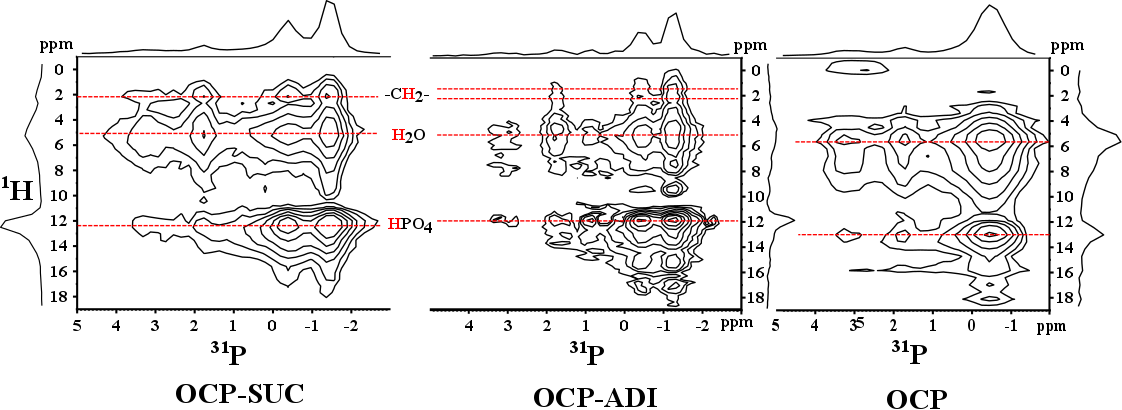


**Figure S3** ^1^H – ^31^P heteronuclear correlation (HETCOR) data for OCP-SUC, OCP-ADI, and pure OCP.

The ^1^H-^31^P heteronuclear correlation (HETCOR) experiment provides information on the distance between hydrogen and phosphorus atoms, which can be used to identify the phosphorus environments and their relationships to the hydration layers. The HETCOR spectra of the composites show similar features to that of pure OCP. It shows ^1^H-^31^P correlation for hydrogen phosphates, and hydrated orthophosphates. The strong correlation between the hydrogen from water hydrogen phosphate to the Pb and Pa phosphorus confirmed that these two phosphate sites are close to the hydrated layer. The ^1^H chemical shifts (F1 dimension, y-axis) of water and hydrogen phosphate in OCP-SUC are to lower frequency than OCP, consistent with the idea that the hydrogen bonding in the hydrated layer of the composites is weaker than that in OCP.

**REFERENCES**

[1] M. Markovic, B.O. Fowler, W.E. Brown, Octacalcium Phosphate Carboxylates. 2. Characterization and Structural Considerations., Chem. Mater. 5 (1993) 1406-16.

[2] Y.H. Tseng, J. Zhan, K.S. Lin, C.Y. Mou, J.C. Chan, High resolution 31P NMR study of octacalcium phosphate, Solid State Nucl. Mag. Reson. 26 (2004) 99-104.

[3] E. Davies, M.J. Duer, S.E. Ashbrook, J.M. Griffin, Applications of NMR crystallography to problems in biomineralization: refinement of the crystal structure and 31P solid-state NMR spectral assignment of octacalcium phosphate, J. Am. Chem. Soc. 134 (2012) 12508-15.

[4] T.W. Tsai, F.C. Chou, Y.H. Tseng, J.C. Chan, Solid-state P-31 NMR study of octacalcium phosphate incorporated with succinate, Phys. Chem. Chem. Phys. 12 (2010) 6692-7.

[5] M. Mathew, W.E. Brown, L.W. Schroeder, B. Dickens, Crystal structure of octacalcium bis(hydrogenphosphate) tetrakis(phosphate) pentahydrate, Ca_8_(HPO_4_)_2_(PO_4_)_4_. 5H_2_0, J. Crystallograph. Spectroscop. Res. 18 (1988) 235-50.
